# pH-dependent stability of honey bee (*Apis mellifera*) major royal jelly proteins

**DOI:** 10.1038/s41598-019-45460-0

**Published:** 2019-06-21

**Authors:** Carmen I. Mureşan, Anja Buttstedt

**Affiliations:** 1 Institut für Biologie, Zoologie - Molekulare Ökologie, Martin-Luther-Universität Halle-Wittenberg, Hoher Weg 4, 06120 Halle (Saale), Germany; 2 Facultatea de Zootehnie şi Biotehnologii, Universitatea de Ştiinţe Agricole şi Medicină Veterinară, Calea Mănăştur 3-5, 400372 Cluj-Napoca, Romania; 3 B CUBE – Center for Molecular Bioengineering, Technische Universität Dresden, Tatzberg 41, 01307 Dresden, Germany

**Keywords:** Protein folding, Proteins, Entomology

## Abstract

Honey bee larval food jelly is a secretion of the hypopharyngeal and mandibular glands of young worker bees that take care of the growing brood in the hive. Food jelly is fed to all larvae (workers, drones and queens) and as royal jelly to the queen bee for her entire life. Up to 18% of the food jelly account for proteins the majority of which belongs to the major royal jelly protein (MRJP) family. These proteins are produced in the hypopharyngeal glands at a pH value of 7.0. Before being fed to the larvae, they are mixed with the fatty acids secreted by the mandibular glands of the worker bees resulting at a pH of 4.0 in the food jelly. Thus, MRJPs are exposed to a broad pH range from their site of synthesis to the actual secreted larval food. We therefore determined the pH-dependent stability of MRJP1, MRJP2 and MRJP3 purified from royal jelly using differential scanning fluorimetry. All MRJPs were much more stable at acidic pH values compared to neutral ones with all proteins showing highest stability at pH 4.0 or 4.5, the native pH of royal jelly.

## Introduction

Honey bees (*Api*s sp.) feed their growing larvae with a special food jelly, a secretion produced by the hypopharyngeal and mandibular glands of nurse worker bees that take care of the brood in the hive^1,2^. The food jelly provides all nutrients that are necessary to develop into an adult and is particularly rich in proteins (11–18% *w*/*w*)^3–5^. Up to 90% of the total proteins belong to the major royal jelly protein (MRJP) family^6^ which comprises ten different proteins (MRJP1-10)^7,8^. However, only MRJP1-3 and 5 are secreted in larger amounts into the food jelly^6^. As a peculiarity, MRJP1 can occur as a monomer (monoMRJP1) or as an oligomer in complex with apisimin (oligoMRJP1/apisimin)^9–12^. The functions of MRJPs reach from providing essential nutrients^6^ over having antibacterial effects^13,14^ and binding RNA^15^ to increasing the viscosity of the food jelly by fibril formation of oligoMRJP1/apisimin at acidic pH 4.0^16,17^. MRJPs share a high amino acid sequence similarity, and thus pH might also play an important role for the other MRJPs as they do indeed encounter a large pH range in the course of their lifetime: The proteins are synthesized in the hypopharyngeal glands of nursing bees^18,19^ as secretory proteins and are thus directly translated into the endoplasmic reticulum of the secretory cells at a pH value of around 7.0^20^. Next, the proteins are stored in secretory vesicles at a pH of 5.5 to 5.1^20–22^. After being secreted from the hypopharyngeal glands, the proteins are exposed to the acidic mandibular gland secretions (pH 3.9 ± 0.1) composed of fatty acids resulting in a final pH of 4.0 in food jelly^21^. Yet MRJPs are by no means limited to the food jelly and can be found in manifold organs and body fluids of honey bees; including the venom^23,24^ (pH 4.5 to 5.2^25,26^), the brain^27,28^ and the hemolymph^29,30^ (pH 6.8^31^). If MRJPs have specific biotic functions they need to be stable within the wide pH range from 4.0 to 7.0 at temperatures normally ranging from 20 °C (bee cluster in winter at outside temperatures of down to −10 °C) to 35 °C (bee cluster during brood rearing)^32^. In addition, extreme temperatures reach from 6 °C (bee in the periphery of the winter cluster)^33^ to 46 °C (bees with activated flight muscles attacking a wasp)^34^. These extreme temperatures might be irrelevant for MRJPs in the food jelly, as the brood is raised at constant temperatures of 34 °C^32^, but might become important for MRJPs being present in the bee venom, the brain and the hemolymph. In addition, most proteins do have their stability optimum at a pH of 7.0 or 7.5 and only very few proteins are stable at a pH below 4.5^35^. However, exactly at these acidic pH values MRJPs need to be stable as they reach their highest incidence in royal jelly at pH 4.0.

The stability of proteins can be assessed by experimentally denaturing their native structure^36^. Transitions between the native and the disordered state are typically induced by denaturing agents, e.g. guanidinium chloride or heat. We determined the melting temperature (*T*m), where the fraction of folded and unfolded protein is equal^36,37^, of purified oligoMRJP1/apisimin, monoMRJP1, MRJP2 and MRJP3 in terms of their pH-dependent stability using differential scanning fluorimetry (ThermoFluor)^37–39^. All tested MRJPs exhibited within their natural pH range (4.0 to 7.0) *T*m values (≥43 °C) above the maximum temperature of 35 °C normally occurring in the hive. In addition, all tested MRJPs were much more stable at pH 4.0, the native pH of the food jelly, compared to pH 7.0 (Δ*T*m = 14.3–18.6 °C) ensuring that MRJPs in the food jelly are not denatured by heat even at elevated ambient temperatures.

## Results

**Figure 1:**
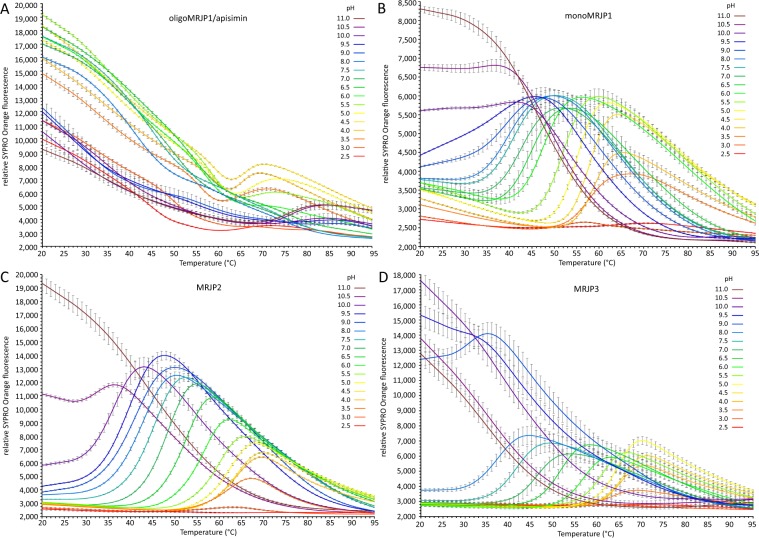
Thermal unfolding curves determined with differential scanning fluorimetry. Measurements were performed at 2 µM protein concentration and 1:1000 SYPRO Orange in four replicates. Curves are exemplarily only shown for one RJ. Measurements from pH 2.5 to 8.0 were performed in 50 mM Na_2_HPO_4_/citric acid and measurements from pH 9.0 to 11.0 in 50 mM Na_2_CO_3_/NaHCO_3_. (**A**) oligoMRJP1/apisimin. (**B**) monoMRJP1. (**C**) MRJP2. (**D**) MRJP3 isoforms.

pH dependent stability of oligoMRJP1/apisimin, monoMRJP1, MRJP2 and the MRJP3 isoforms was determined covering a pH range from 2.5 to 11. At the beginning of the experiment, at low temperature, a low fluorescence is expected as an indicator of a well folded protein. Within the physiological pH range for MRJPs (pH 4.0–7.0), monoMRJP1, MRJP2 and the MRJP3 isoforms possess at 20 °C a rather low fluorescence around 3,000–3,500 relative fluorescence units (RFU) (Fig. 1B–D). Contrary to that, the complex of oligoMRJP1/apisimin exhibited at the same pH values already at 20 °C a fluorescence between 15,000–20,000 RFU (Fig. 1A) indicating exposed hydrophobic regions. For pH 4.0 to 6.0, this fluorescence declines continuously until approximately 60 °C, followed by a further fluorescence increase peaking between 67 to 74 °C. For pH 6.5 and 7.0, the decline in fluorescence was constant. In addition, MRJP2 has a higher fluorescence intensity increase than monoMRJP1, and MRJP3 within the physiological pH range (Δfluorescence: monoMRJP1, ~3,000; MRJP2, ~10,000; MRJP3, ~4,000) implying a tighter packed hydrophobic core for MRJP2.

At pH 2.5 and 3.0, the curves recorded for monoMRJP1, MRJP2 and MRJP3 did either not show any increase in fluorescence at all or just a very slight one of approximately 500 RFU (Fig. 1B–D). Either the proteins do not unfold at these acidic pH values or the increased solvent polarity at decreased pH values leads to lower fluorescence intensity or shifting of the maximum fluorescence emission to other wavelengths as shown for other fluorescent dyes than SYPRO Orange^40^. Again, the complex of oligoMRJP1/apisimin exhibited also at these pH values an increased starting fluorescence (~10,000–12,000 RFU) which decreased at elevated temperatures (Fig. 1A).

At pH values above 9.0, starting fluorescence gradually increased with higher pH also for monoMRJP1, MRJP2 and MRJP3 indicating exposed hydrophobic residues and protein unfolding even at lower temperatures (monoMRJP1 pH ≥10.0, MRJP2 pH ≥10.5, MRJP3 ≥9.0).

Due to the increased starting fluorescence for oligoMRJP1/apisimin, transition midpoints (*T*m) were only determined for monoMRJP1, MRJP2 and MRJP3. All proteins were much more stable at pH 4.0 than at pH 7.0 (Δ*T*m: 14.3–18.6 °C) (Table 1; Fig. S2) (Kruskal-Wallis ANOVA (n = 360; H = 351; *P* < 0.001), monoMRJP1: *P* = 0.016; MRJP2: *P* = 0.004; MRJP3: *P* < 0.001) and all MRJPs showed maximal thermal stability around the native pH of the food jelly (4.0–4.5). Therefore, monoMRJP1, MRJP2 and MRJP3 belong to the ~5% of proteins that have their pH-optimum of stability at pH 4.5 or less^35^.

**Table 1:** Transition midpoints (*T*m) of the differential scanning fluorimetry unfolding curves.

pH	monoMRJP1	MRJP2	MRJP3
3.5	58.6 ± 0.7 °C^a,b^	61.3 ± 1.3 °C^a,b^	62.4 ± 0.4 °C^a,b^
4.0	**59**.**3** ± **0**.**5** °**C**^**a**^	63.2 ± 1.3 °C^a^	64.2 ± 0.3 °C^a^
4.5	58.0 ± 0.6 °C^a,b^	**63**.**6** ± **1**.**3** °**C**^**a**^	**64**.**8** ± **0**.**4** °**C**^**a**^
5.0	55.4 ± 0.6 °C^a–c^	62.3 ± 1.3 °C^a^	63.5 ± 0.3 °C^a^
5.5	52.2 ± 0.9 °C^a–c^	59.7 ± 1.4 °C^a–c^	60.1 ± 0.2 °C^a,b^
6.0	48.5 ± 1.3 °C^a–c^	56.4 ± 1.3 °C^a–d^	56.1 ± 0.1 °C^a–c^
6.5	45.7 ± 1.4 °C^a–c^	52.8 ± 1.4 °C^a–d^	50.8 ± 0.2 °C^b–d^
7.0	43.3 ± 2.4 °C^b,c^	48.9 ± 1.5 °C^b–d^	45.6 ± 0.3 °C^c,d^
7.5	40.9 ± 2.1 °C^c^	46.0 ± 1.6 °C^c,d^	41.5 ± 0.4 °C^c,d^
8.0	39.3 ± 1.7 °C^c^	43.3 ± 1.9 °C^d^	38.1 ± 1.3 °C^d^
9.0	36.1 ± 1.3 °C	42.8 ± 1.7 °C	largely unfolded
9.5	largely unfolded	40.8 ± 1.8 °C	unfolded
10.0	unfolded	37.0 ± 1.4 °C	unfolded
10.5	unfolded	largely unfolded	unfolded
11.0	unfolded	unfolded	unfolded

Values are means ± standard deviations (3 biological replicates (proteins purified from three different royal jellies), 4 technical replicates per protein). Statistics were performed only from pH 3.5 to 8.0 where values for all three proteins were present (Kruskal-Wallis ANOVA, n = 360; H = 351; p < 0.001). ^a–d^*T*ms in the same row with different superscripts are significantly different (P < 0.05). The highest *T*m for each protein is highlighted in bold.

## Discussion

Transition midpoints could be determined for all proteins except for the complex of oligoMRJP1 and apisimin, which showed elevated starting fluorescence already at low temperatures indicating exposed hydrophobic regions. As monoMRJP1 does not show any peculiarity regarding hydrophobicity, the high hydrophobicity of oligoMRJP1/apisimin might be attributed to several reasons: (1) Either to the bound apisimin itself, which consists of ~40% hydrophobic amino acids, or (2) to hydrophobic residues of MRJP1 which are exposed only after apisimin binding. Hydrogen/deuterium exchange experiments indeed revealed that the N-terminal part of MRJP1 within oligoMRJP1/apisimin is highly disordered^11^ but a comparison to the structure of monoMRJP1 is missing. (3) Only very recently it has been revealed that the complex of oligoMRJP1 and apisimin binds in addition eight molecules of 24-methylenecholesterol^41^ which explains most likely the high hydrophobicity. However, also a combination of the reasons mentioned might be possible. Still, the melting curves of oligoMRJP1/apisimin have marked transitions points at pH values between 4.0 and 6.0 (*T*m ~ 66–70 °C) which might be caused by the unfolding of the structured part of the complex. OligoMRJP1/apisimin (pH 4.0–6.0; *T*m ~ 66–70 °C) appears to have an increased stability compared to monoMRJP1 (pH 4.0–6.0; *T*m = 59.3–48.5 °C), irrespective of the fact that some hydrophobic residues are already exposed at low temperatures. At neutral pH, fluorescence of oligoMRJP1/apisimin constantly decreased and no evaluation of the curves was possible in this study. However, it was shown that incubation of oligoMRJP1/apisimin in phosphate buffered saline (pH 7.5) at 56 °C for 30 min did not lead to a dissociation of the complex^42^ and that the heat treatment did not impair proliferation activity of the complex on human lymphoid cells^43^. Circular dichroism spectroscopy in 2 mM HEPES, pH 7.0 showed even a slight structural gain from 20 °C to 95 °C^44^. This suggests that oligoMRJP1/apisimin is still folded at elevated temperatures at near neutral pH and illustrates that differential scanning fluorimetry is not an appropriate method to analyze the stability of the complex of oligoMRJP1 and apisimin. In addition, no strong difference was observed between fluorescence curves recorded above and below pH 5.0 although oligoMRJP1/apisimin starts to assemble into fibrillary structures below pH 5.0^16,17^.

MonoMRJP1, MRJP2 and the MRJP3 isoforms were all much more stable at pH 4.0 than at neutral pH (Δ*T*m: 14.3–18.6 °C). At elevated alkaline pH (≥9.0), hydrophobic residues were already exposed at 20 °C, residues which were buried at lower pH values. Indeed MRJPs have been shown to be more stable against limited proteolysis at acidic rather than alkaline conditions^45^. The different MRJPs showed at pH 4.0 a remarkable stability with melting temperatures ranging between 59.3 to 64.2 °C. This is in accord with accelerated protein degradation in fresh RJ above 65 °C^46^ as the proteins unfold at these temperatures.

Minimum solubility often coincides with the isoelectric point (pI) of proteins^47^ where the electrostatic forces are at minimum and the proteins might precipitate out of solution. MRJPs exhibit theoretical pIs between 5.0 and 6.7 (monoMRJP1 and oligoMRJP1/apisimin–5.0, MRJP2–6.7, MRJP3–6.5) and should thus show lowest solubility at a pH of 5.0 (monoMRJP1 and oligoMRJP1/apisimin) and ~6.5 (MRJP2 and MRJP3). In the worker bee, all MRJPs reach their highest concentration in the hypopharyngeal gland secretion, before being mixed with the mandibular gland secretion to produce the final food jelly. Interestingly, the hypopharyngeal gland secretion has a pH of 5.1 ± 0.1^21^, which is exactly at the pI of monoMRJP1 and oligoMRJP1/apisimin. Thus, the increased protein stability at the acidic pH values is essential to ensure that MRJPs, and especially oligoMRJP1/apisimin, do not precipitate in the hypopharyngeal gland secretion as this would lead to blocking of the glands and prevent further food jelly secretion.

## Materials and Methods

### Royal jelly samples and protein purification

Three fresh royal jelly (RJ) samples (*Apis mellifera*), used for protein purification, were acquired in 2015 from three different beekeepers in Romania (RJ1 - Cluj-Napoca, Cluj County – July/RJ2 - Bratca, Bihor County – August/RJ3 - Cluj-Napoca, Cluj County - June). All RJ samples were stored at −20 °C. OligoMRJP1/apisimin, monoMRJP1 and MRJP2 were purified via cation exchange chromatography using SP Sepharose Fast Flow (GE Healthcare, Little Chalfont, UK) according to previous studies^45,48^. MRJP3 and 5 that co-eluted from the SP Sepharose column with 50 mM Na_2_HPO_4_/NaH_2_PO_4_, 200 mM NaCl, pH 7.0 were rebuffered into 50 mM Na_2_HPO_4_/NaH_2_PO_4_, 2 M (NH_4_)_2_SO_4_, pH 7.0 using PD-10 columns (GE Healthcare). The rebuffered sample (~2 ml) with an absorption at 280 nm of maximum 4.5, as measured with a NanoDrop ND-1000 Spectrophotometer (Thermo Fisher Scientific, Waltham, MA, USA), was loaded onto a 1 ml Butyl-Sepharose 4 Fast Flow (GE Healthcare) column. The column was washed with 10 ml 50 mM Na_2_HPO_4_/NaH_2_PO_4_, 2 M (NH_4_)_2_SO_4_, pH 7.0 and elution of MRJP3 was achieved with 10 ml 50 mM Na_2_HPO_4_/NaH_2_PO_4_, 0.75 M (NH_4_)_2_SO_4_, pH 7.0. Fractions of 1 ml were collected and protein purity was verified with sodium dodecyl sulfate (SDS) polyacrylamide gel electrophoresis (PAGE)^49^ and native PAGE at pH 7.0^50^. Pure protein fractions were combined (Fig. S1).

Due to a repetitive region with length polymorphisms^51^, MRJP3 occurs in different isoforms showing as multiple bands on SDS PA gels between 60 and 70 kDa^15,45^ (Fig. S1, lane 6). Buffer exchange for further experiments was performed with PD-10 desalting columns. Protein concentrations were determined via UV spectroscopy. Molar extinction coefficients were calculated with ProtParam^52^ as 56,185 M^−1^ cm^−1^ for oligoMRJP1/apisimin and monoMRJP1, 51,590 M^−1^ cm^−1^ for MRJP2 and 47,580 M^−1^ cm^−1^ for MRJP3.

### Thermal unfolding using ThermoFluor

To monitor the pH dependency of MRJP unfolding, ThermoFluor experiments^37–39^ were conducted. This method is based on monitoring the fluorescence of specific dyes, e.g. Sypro Orange, which is quenched in aqueous solutions but is highly fluorescent in presence of hydrophobic sites of unfolded proteins^37,39^. Thus, upon proceeding protein unfolding, fluorescence intensity increases and can be plotted as function of temperature. Experiments were performed in Hard-Shell 96-well microplates sealed with Microseal ‘B’ seals in a CFF Connect Real-Time System (all Bio-Rad, Hercules, CA, USA). Measurements were performed (3 biological replicates (proteins purified from three different royal jellies), 4 technical replicates per protein) in a total volume of 20 µl with 2 µM oligoMRJP1/apisimin, monoMRJP1, MRJP2 and MRJP3 and SYPRO Orange (Sigma-Aldrich, St. Louis, MO, USA) at a dilution of 1:1000 in 50 mM Na_2_HPO_4_/citric acid pH 2.5–8.0 or 50 mM Na_2_CO_3_/NaHCO_3_ pH 9.0–11.0. Samples were heated from 20 °C to 95 °C at 1 °C per min and fluorescence intensity of SYPRO Orange was measured every degree using the FRET channel of the Real-Time System (excitation: 450–490 nm, detection: 560–580 nm). To determine the melting temperature (*T*m - temperature at which the concentrations of folded and unfolded protein are equal), fluorescence intensities (FI) were plotted as a function of temperature (T) and fitted with Origin 5.0 (Microcal Software Inc., Northampton, MA, USA) according to the Boltzmann equation.

$$FI=F{I}_{MIN}+\frac{(F{I}_{MAX}-F{I}_{MIN})}{1+\exp (\frac{Tm-T}{dT})}.$$

## Supplementary information

Supplementary Information
