# Supplementary material for: pH-dependent stability of honey bee (Apis mellifera) major royal jelly proteins
Source: Sci Rep. 2019 Jun 21;9:9014. doi: 10.1038/s41598-019-45460-0 (PMC6588556; doi:10.1038/s41598-019-45460-0)
Supplement: Supplementary file 1 — Supplementary Information [file 41598_2019_45460_MOESM1_ESM.pdf]

Supplementary Information

for

pH-dependent stability of honey bee (*Apis mellifera*) major royal jelly proteins

Carmen I. Mureşan & Anja Buttstedt

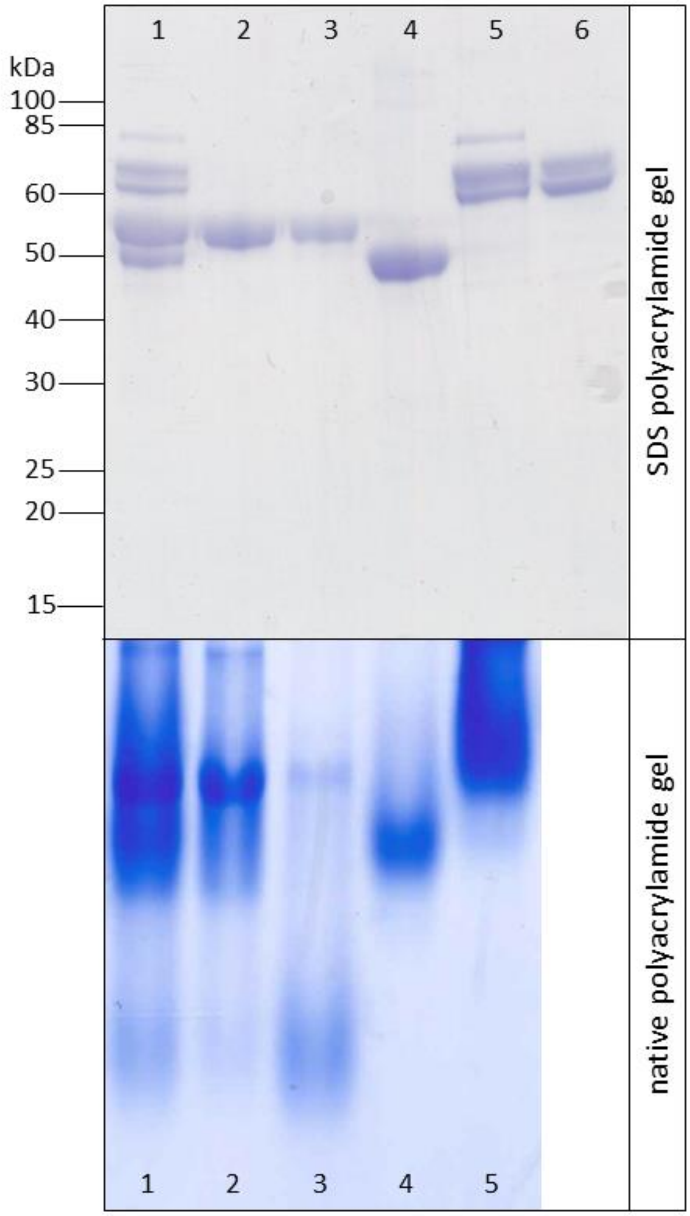

Figure S1: SDS and native polyacrylamide gel exemplarily showing MRJP purification for one RJ. 1, Royal jelly protein extract; 2, oligoMRJP1/apisimin; 3, monoMRJP1; 4, MRJP2; 5, MRJP3 isoforms and MRJP5 co-eluting, 6, MRJP3 isoforms. The MRJP3 double band arises due to the fact that MRJP3 contains a repetitive region with varying repeats between isoforms[51]. With the current methods, it is not possible to separate the individual isoforms from each other and the MRJP3 isoforms are usually investigated together[45,15]. The lower gel shows a native PA gel. Here, no SDS is added and the proteins are separated depending on their individual charge. MRJPs are known to vary in pI due to differential posttranslational modifications and this causes the smear in some of the lanes. Figure S3 shows the gels that were not cropped.

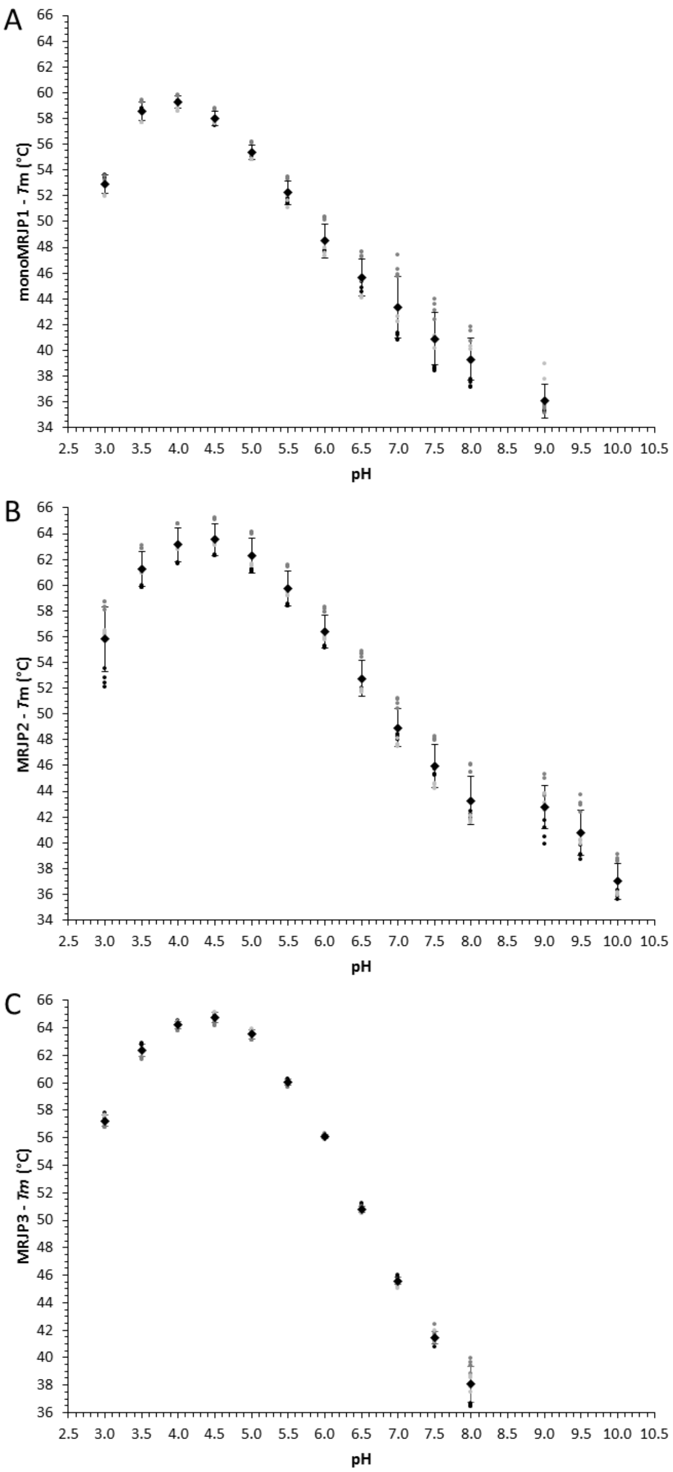

Figure S2: Differential scanning fluorimetry. Transition midpoints of the thermal unfolding of monoMRJP1 **(A)**, MRJP2 **(B)** and MRJP3 **(C)** purified from three different RJs (black dots - RJ1, dark grey dots - RJ2, light grey dots - RJ3, black diamonds - midpoints averaged from the three RJs ± standard deviation). The proteins were measured at a concentration of 2 µM in 50 mM Na_2HPO_4/citric acid from pH 3.0 to 8.0 and in in 50 mM Na_2CO_3/$NaHCO_3$ from pH 9.0 to 10.0.

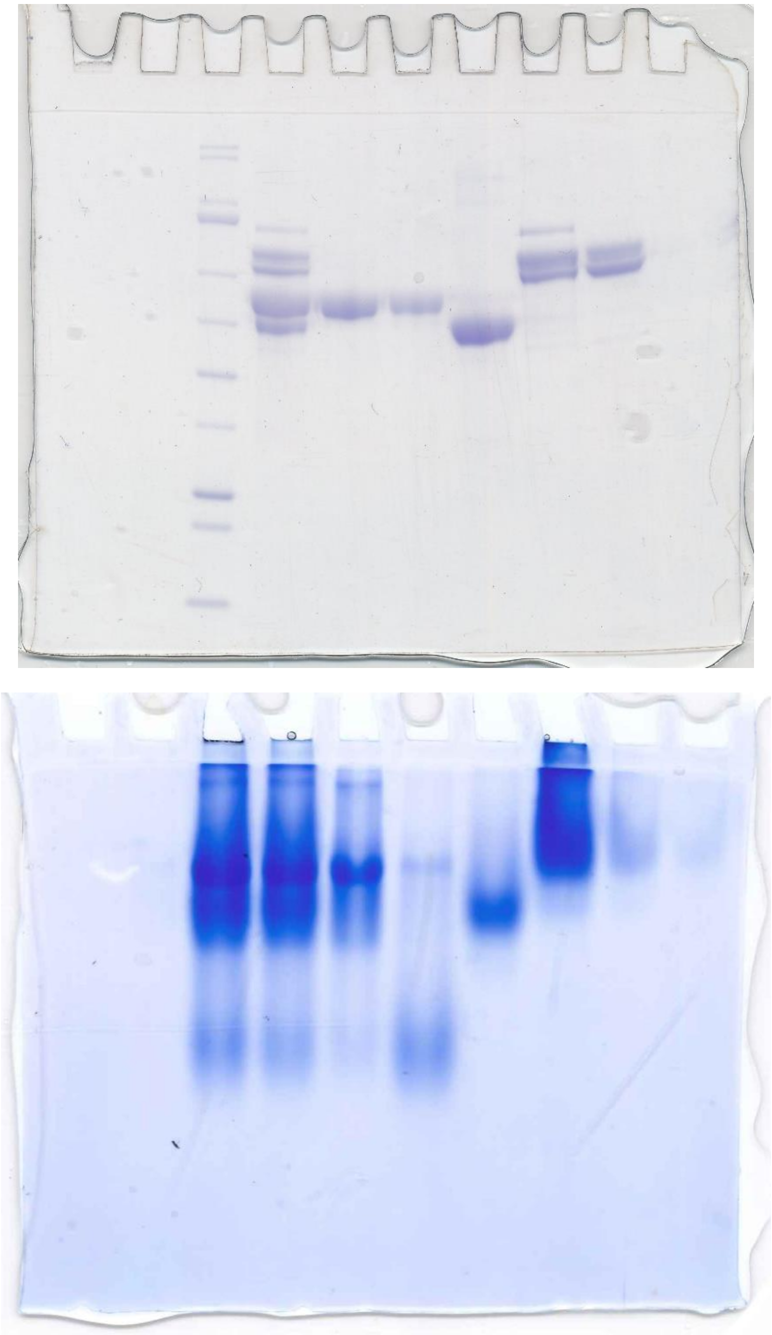

Figure S3: Full-length gels of Figure S1.
